# A Flexible Method to Fabricate Exsolution‐Based Nanoparticle‐Decorated Materials in Seconds

**DOI:** 10.1002/advs.202200250

**Published:** 2022-02-20

**Authors:** Zhu Sun, Weiwei Fan, Yu Bai

**Affiliations:** ^1^ State Key Laboratory of Electrical Insulation and Power Equipment Xi'an Jiaotong University Xi'an 710049 P. R. China; ^2^ Department of Nuclear Science and Engineering Massachusetts Institute of Technology Cambridge MA 02139 USA; ^3^ State Key Laboratory for Mechanical Behavior of Materials Xi'an Jiaotong University Xi'an 710049 P. R. China

**Keywords:** exsolution, nanoparticle‐decorated material, solid oxide cell, thermal shock

## Abstract

Decorating metallic nanoparticles on the surface of oxide support is a promising approach to tailor the catalytic activity of perovskite. Here, for the first time using thermal shock to rapidly fabricate nanoparticle‐decorated materials (NDMs) is proposed. Low‐cost and size‐tailorable carbon paper is used as the heating source during the thermal shock. It is reported that by thermal shock technique, only ≈13 s including heating and treating time is needed to fabricate the exsolution‐based NDMs (the fastest method to date). Benefitted by the sufficiently provided driving force and the short treating time, as compared to the product prepared by the conventionally furnace‐based method, higher particle density and smaller particle size of the exsolved catalysts are acquired for the thermal shock fabricated NDM, giving rise to a fascinating improvement (12‐fold) of the electrochemical performance. This work develops a new technique to rapidly fabricate NDMs in an economic and high‐throughput manner, profoundly improving the flexibility of the application of exsolution‐based materials in electrochemical devices.

## Graphical abstract

By thermal shock technique, exsolution‐based nanoparticle‐decorated materials (NDMs, the fastest method to date) can be fabricated in seconds. As compared to the product prepared by the conventionally furnace‐based method, higher particle density and smaller particle size of the exsolved catalysts are obtained for the thermal shock fabricated NDM, giving rise to a fascinating improvement of the electrochemical performance.

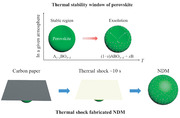

## 1 Introduction

Exsolution is a very promising approach to tailor the electrocatalytic activity of the perovskite material,^[^ ^1^, ^2^, ^3^, ^4^, ^5^, ^6^ ^]^ primarily benefitted by the increased active sites.^[^ ^7^, ^8^, ^9^, ^10^, ^11^, ^12^ ^]^ Briefly, transition metal(s) with good catalytic activity is incorporated into the perovskite lattice to form a solid solution in oxidizing environment, and followed by exsolution from the host lattice through reducing treatment. Different from the deposition method to fabricate the nanoparticle‐decorated materials (NDMs),^[^ ^13^, ^14^ ^]^ the products prepared by exsolution technique generally exhibit good spatial distribution and thermal stability due to the strong bonding between nanoparticle and support.^[^ ^15^, ^16^, ^17^, ^18^, ^19^, ^20^, ^21^ ^]^ Therefore, exsolution‐based NDMs are broadly applied in all‐solid electrochemical cells.^[^ ^11^, ^18^, ^22^, ^23^ ^]^

Conventionally, exsolution‐based NDMs are usually prepared via chemical gas treatment.^[^ ^20^, ^24^ ^]^ Because of the sluggish diffusion of ions traversing from bulk to the surface of the perovskite support, normally tens of hours are needed to obtain the NDM products. As a result, the size of the exsolved nanoparticles is usually large due to the overlap with neighboring particles during the long‐time treatment, resulting in an underestimated improvement of the electrocatalytic activity by this technique. To accelerate the exsolution processes, recently, an electrochemical poling approach was reported, which could rapidly induce the exsolution.^[^ ^2^ ^]^ For the previously reported methods, they generally relied on a conventional furnace to accomplish the exsolution. In this case, one usually needs to wait for several hours to achieve to the desired temperature due to the low heating rate restricted by the heating elements of the furnace. Moreover, for the electrochemically induced exsolution,^[^ ^2^ ^]^ the exsolution only could be realized on an as‐prepared cell at high temperatures. This is mainly because that at low temperatures, the resistance of the electrolyte will increase, at that time the potential distributed on the electrode will not be sufficient to induce the exsolution. Moreover, at one time, only one cell could be treated, limiting the fabrication of NDMs in a high‐throughput manner.

**Figure 1:**
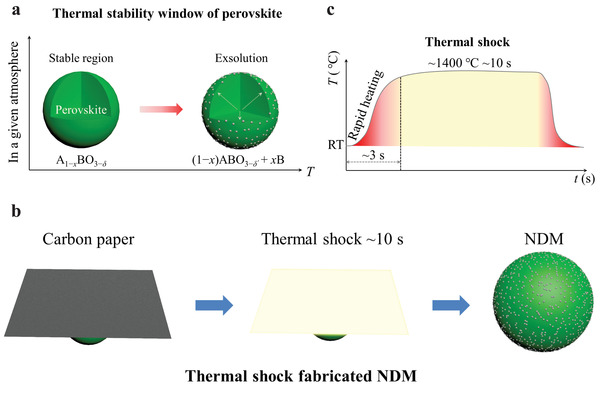
a) Thermal stability window of perovskite under a given atmosphere. b) Illustration of the thermal shock fabricated nanoparticle‐decorated material (NDM). c) Temperature profile for the thermal shock technique.

Generally, exsolution could take place under the occurrence of phase decomposition of the parent perovskites and/or generation of oxygen vacancies without structure decomposition.^[^ ^25^, ^26^, ^27^ ^]^ Moreover, for different types of perovskites, the driving force required to induce the exsolution is different due to the different reducibility.^[^ ^28^, ^29^ ^]^ From thermodynamics and kinetics points of view, to trigger the exsolution from perovskite oxides, the driving force can be provided by the thermal potential (*T*). More specifically, for a certain perovskite, there should exist a thermal stability window under a given atmosphere (**Figure** **1**a). Within the stable region, exsolution cannot take place. However, if the driving force supplied to the system exceeds the energy barrier of the exsolution, exsolution will dynamically occur. It could be expected that by rapidly increasing the thermal potential gradient between the perovskite oxide and environment, the exsolution kinetics would be greatly enhanced.^[^ ^30^ ^]^ Generally, the diffusivity of active species follows the Arrhenius relation *D* = *D*_0_exp(−*Q*/*k*_B_ *T*), hence elevated temperature facilitates the ion diffusion and cluster nucleation. Moreover, one could tailor the particle size through adjusting the treatment time at elevated temperature. Besides, to leverage the potential source of driving force for inducing exsolution, chemical gas could be introduced to the system. Therefore, there stands a good chance to acquire exsolution‐based NDM with higher particle density and smaller particle size in a shorter time by treating the perovskite oxide at elevated temperature in reducing atmosphere. Higher particle density and smaller particle size would provide more surface area and extend the length of triple phase boundary,^[^ ^7^ ^]^ thus supplying more active sites for the electrochemical reactions. Eventually, a further improved catalytic activity could be anticipated.

With the aim of rapidly fabricating exsolution‐based NDMs in an economic and high‐throughput manner, herein we proposed a new approach, named thermal shock, to trigger the exsolution from perovskite oxide (Figure 1b). To demonstrate our concept, perovskite La_0.43_Ca_0.37_Ti_0.94_Ni_0.06_O_3−_ *_*δ*_* (LCTN) was applied as a model. Low‐cost and size‐tailorable carbon paper was used as the heating source and placed close to the treated sample. When a customized current was supplied to the carbon paper, a lot of Joule heating was generated and the temperature rapidly went up to ≈1400 ± 100 °C in ≈3 s (Figure 1c). It was found that after thermal shock for ≈10 s in 5%H_2_/N_2_ atmosphere, numerous nanoparticles with good distribution appeared on the surface of LCTN. Namely, only around 13 s (including heating and treating time), the fastest method as far as we know, was needed to fabricate the exsolution‐based NDMs. As compared to the product prepared by the conventionally furnace‐based approach (900 °C, 20 h), higher particle density and smaller particle size were obtained by the thermal shock technique, giving rise to a better electrochemical performance. Thermal shock technique profoundly enhances the efficiency and flexibility to fabricate the exsolution‐based NDMs.

## 2 Results and Discussion

### 2.1. Microstructure of LCTN Perovskite after Thermal Shock

In this work, La_0.43_Ca_0.37_Ti_0.94_Ni_0.06_O_3−_ *_*δ*_* (LCTN) oxide was prepared by solid‐state reaction method and used as a model. Generally, a much more dynamic exsolution could be expected by changing the equilibrium position, therefore, modest A‐site deficiency was introduced into the target material.^[^ ^1^ ^]^ After Rietveld refinement, it was found that the as‐prepared LCTN oxide could be commendably indexed on the basis of an orthorhombic structure (space group *Pbnm* (62)). Namely, BO_6_ octahedrons are filled with A‐site atoms at the interstitial spaces (Figures S1 and S2, Supporting Information). The lattice parameters are *a* = 5.4643(4) Å, *b* = 7.7337(4) Å, and *c* = 5.4632(7) Å (Table S1, Supporting Information).

**Figure 2:**
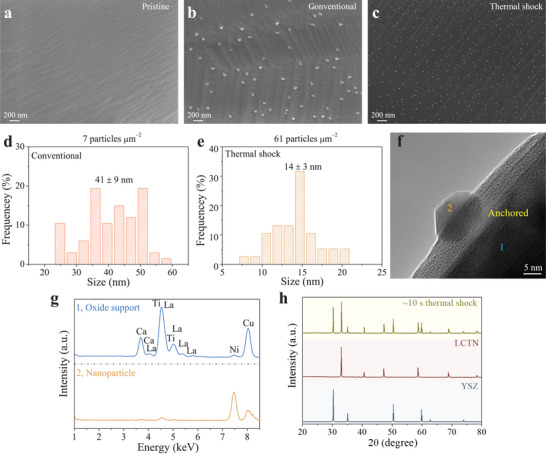
a) SEM image of the pristine LCTN. b) SEM image of the LCTN after gas treatment in 5%H_2_/N_2_ at 900 °C for 20 h. c) SEM image of the LCTN after thermal shock in 5%H_2_/N_2_ for ≈10 s. d,e) Corresponding particle size distribution of figure (b) and (c). f) TEM image of the exsolved particle after thermal shock. g) TEM‐EDS results of the exsolved nanoparticle and oxide support. h) XRD pattern of LCTN after thermal shock. For comparison, YSZ and LCTN profiles are also shown.

To study the influence of gas treatment and thermal shock on the exsolution of nanoparticles from perovskite oxide support, the as‐synthesized LCTN materials were divided into three batches, and then the corresponding treatments were conducted on the samples. Scanning electron microscopy (SEM) was applied to investigate the microstructure of the samples before and after treatment. As shown in **Figure** **2**a, the pristine LCTN presented a terrace‐like structure, which is typical for the A‐site‐deficient perovskite oxide. Meanwhile, the surface was neat and no particles were observed on the oxide support. Subsequently, the LCTN was placed in a tube furnace and heated up to 900 °C at heating rate of 5 °C min^−1^ in 5%H_2_/N_2_ atmosphere. It was found that even after the chemical gas treatment for 20 h, only a small number of nanoparticles formed on the surface of LCTN support (Figure 2b). This is mainly due to the weak driving force provided by the chemical gas, giving rise to slow exsolution kinetics as well as “low‐activated” surface, limiting the cluster nucleation. When a carbon paper was used as the heating source, a much higher heating rate of ≈10^4^ °C min^−1^ was attained. Surprisingly, after thermal shock at ≈1400 °C for ≈10 s in 5%H_2_/N_2_ atmosphere, impressive nanoparticles appeared on the surface of LCTN (Figure 2c and Figure S3, Supporting Information), implying that the exsolution kinetics was enhanced significantly by thermal shock. In fact, during the thermal shock, apart from the weak driving force supplied by the chemical gas, the extremely high temperature could provide extraordinary driving force to the system, prominently accelerating the exsolution of nanoparticles from the perovskite lattice.

Moreover, results showed that for conventional chemical gas induced exsolution, the population density was ≈7 particles µm^−2^, while the average particle size was 41 ± 9 nm (Figure 2d). However, regarding the thermal shock‐triggered exsolution, the population density greatly increased to ≈61 particles µm^−2^, bringing about an improved concentration of active sites for electrochemical reactions. Concurrently, the average particle size decreased to 14 ± 3 nm (Figure 2e). Generally, the eventual particle density is related to the nucleation sites existing and additionally generated on the surface of the perovskite support.^[^ ^31^, ^32^ ^]^ The strong driving force supplied by the thermal shock kinetically facilities the formation of nucleation sites, such as oxygen vacancies, and thus increasing the concentration of the nucleation sites. Moreover, the generated metal could be used as the feeding source to sustain the particle growth. Due to the short treating time and increased concentration of the nucleation sites, a smaller particle size was eventually acquired.

As a reference, La_0.43_Ca_0.37_TiO_3−_ *_*δ*_* (LCT) perovskite (Figure S4, Supporting Information) without exsolvable element was also prepared and treated as the same routine of LCTN. It was found that no nanoparticles formed on the surface of LCT oxide support after thermal shock (Figure S5, Supporting Information), suggesting an exsolution of Ni from LCTN perovskite after thermal shock. To get more information about the microstructure, subsequently, transmission electron microscopy (TEM) was carried out. As shown in Figure 2f, it was observed that the exsolved nanoparticle partially submerged in the oxide support. In this architecture, a strong interaction between nanoparticle and support can be expected.^[^ ^5^, ^15^, ^33^, ^34^ ^]^ Moreover, TEM‐EDS (energy‐dispersive X‐ray spectroscopy) result showed that the intensity of Ni improved remarkably (Figure 2g), while it decreased for the rest elements, indicating the exsolution of Ni after thermal shock. In addition, a lattice spacing of 0.269 nm was obtained for the exsolved nanoparticle after thermal shock (Figure S6, Supporting Information), which was in good accordance with the (111) plane of Ni phase.

Additionally, X‐ray diffraction (XRD) was conducted on the LCTN after thermal shock. For comparison, the XRD profiles of YSZ and as‐prepared LCTN were also shown. Results showed that, apart from the diffraction peaks coming from the YSZ electrolyte, the rest peaks of the LCTN after thermal shock matched well with the as‐prepared LCTN, suggesting the perovskite crystal structure still maintained after ≈10 s thermal shock treatment, presumably benefited by the short treatment time. Overall, thermal shock is a powerful technique to get the exsolution‐based NDMs with higher particle density and smaller particle size in a captivatingly rapid manner.

### 2.2. Electrocatalytic Activity of the LCTN after Thermal Shock

**Figure 3:**
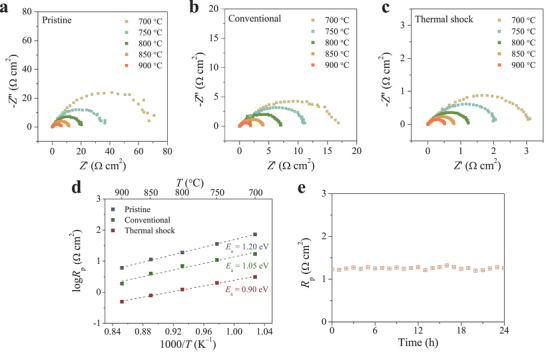
Nyquist plots of the YSZ supported symmetrical cells using a) pristine LCTN, b) conventional exsolution LCTN, and c) thermal shock exsolution LCTN as the electrodes collected from 700 to 900 °C in humidified (≈3% H_2_O) 5%H_2_/N_2_ atmosphere under OCV conditions. d) Arrhenius plots of *R*_p_ for the corresponding electrodes. e) *R*_p_ of the thermal shock exsolution LCTN collected at different time at 800 °C under OCV condition.

To investigate the electrocatalytic activity of the LCTN perovskite, symmetrical cells LCTN|YSZ|LCTN were prepared and the electrochemical impedance spectroscopy (EIS) was collected under open‐circuit voltage (OCV) in 5%H_2_/N_2_ atmosphere by using the home‐made setup (Figure S7, Supporting Information). For the pristine LCTN, the polarization resistance (*R*_p_) decreased from 67 to 6.1 Ω cm^2^ in the temperature range of 700–900 °C (**Figure** **3**a), ascribing to the thermal activation of the electrochemical reactions. The mediocre *R*_p_ values suggested an inferior catalytic activity of the pristine LCTN. Regarding the LCTN after conventional exsolution, lower *R*_p_ values were obtained during the measurements. The *R*_p_ value changed between 1.9 and 17 Ω cm^2^ (Figure 3b), implying an enhancement of the electrocatalytic activity. Exhilaratingly, as to the LCTN after thermal shock‐triggered exsolution, the *R*_p_ value decreased remarkably. In the whole temperature range, it varied from 3 to 0.5 Ω cm^2^ (Figure 3c), indicating a prominent improvement of the electrochemical kinetics. As compared to that of pristine LCTN, almost 22‐fold enhancement was attained at 700 °C. This is primarily due to that the impressively exsolved nanocatalysts could provide numerous active sites, greatly accelerating the corresponding electrochemical reactions.

Generally, activation energy (*E*_a_), associating with the electrochemical processes of gas adsorption, dissociation, and diffusion, is acquired via the equation

(1) $$\log R_{p}\;=\;\log R_{0}\;-\frac{E_{a}}{2.303RT}$$

After calculation, *E*_a_ values of 1.20 and 1.05 eV were acquired for the pristine LCTN and conventional exsolution prepared LCTN. However, a lower *E*_a_ value of 0.90 eV was obtained for the LCTN after thermal shock‐triggered exsolution, further indicating the enhanced catalytic activity. In addition, no obvious change on the *R*_p_ was observed during the following operation (Figure 3e).

### 2.3. Cell Performance

**Figure 4:**
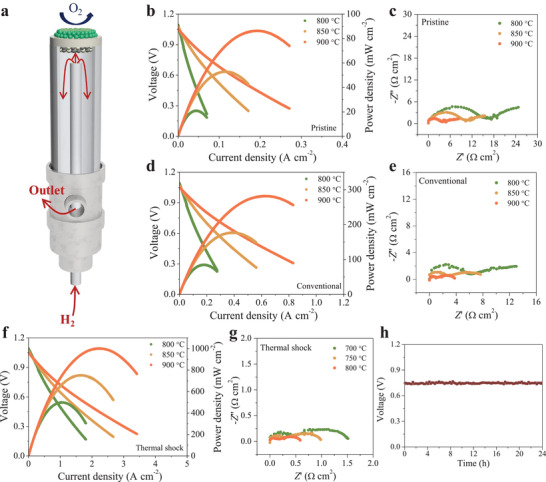
a) Schematic of the home‐made setup for the full cell performance test. b) Voltage and power density versus current density for the cell LCTN(pristine)|YSZ|LSM‐YSZ measured from 800 to 900 °C using humidified (3% H_2_O) H_2_ as fuel and air as oxidant. c) Nyquist plots of the cell LCTN(pristine)|YSZ|LSM‐YSZ collected under OCV conditions. d) Voltage and power density versus current density for the cell LCTN(conventional)|YSZ|LSM‐YSZ. e) Nyquist plots of the cell LCTN(conventional)|YSZ|LSM‐YSZ collected under OCV conditions. f) Voltage and power density versus current density for the cell LCTN(thermal shock)|YSZ|LSM‐YSZ. g) Nyquist plots of the cell LCTN(thermal shock)|YSZ|LSM‐YSZ collected under OCV conditions. h) Cell voltage as a function of time for the cell LCTN(thermal shock)|YSZ|LSM‐YSZ operated under a constant current density of 0.52 A cm^−2^ at 800 °C.

In order to get more information about the electrochemical performance of LCTN perovskite, YSZ electrolyte‐supported cells LCTN|YSZ|LSM‐YSZ were investigated using the home‐made setup (**Figure** **4**a). During the measurements, wet (≈3% H_2_O) hydrogen was used as the fuel gas, while the ambient air was applied as the oxidant.

With respect to the pristine LCTN (Figure 4b,c), at 800 °C, the peak power density (PPD) value was 21 mW cm^−2^ and *R*_p_ value was 25 Ω cm^2^, suggesting a sluggish electrochemical reaction due to the inferior electrocatalytic activity. Derived from the enhanced activity at higher temperatures, higher PPD values of 53 and 86 mW cm^−2^ were attained at 850 and 900 °C, respectively. Concurrently, lower *R*_p_ values of 15 Ω cm^2^ at 850 °C and 8.3 Ω cm^2^ at 900 °C were obtained. For conventional LCTN, the LCTN perovskite was first treated by chemical gas 5%H_2_/N_2_ for 20 h at 900 °C to match with the half‐cell condition. At this point, hydrogen fuel gas was introduced into the anode chamber. After stabilizing, the measurements were conducted from 900 to 800 °C with a temperature interval of −50 °C. Results showed that a commonplace improvement of the electrochemical performance was obtained (Figure 4d,e) for the conventional LCTN treated by the chemical gas. In the temperature range of 800–900 °C, the PPD went from 85 to 281 mW cm^−2^, and the *R*_p_ went from 13 to 3.8 Ω cm^2^. Considering the rest components of the cell were similar, the performance change most probably came from the fuel electrode. Actually, the exsolved nanoparticles could improve the electrocatalytic activity of the fuel electrode on hydrogen oxidation reaction. Fascinatingly, for the LCTN after thermal shock (Figures S8 and S9, Supporting Information), the cell performance was enhanced prominently (Figure 4f,g). The PPD value reached to 1003 mW cm^−2^ at 900 °C, ≈12‐fold enhancement was achieved as compared to that of pristine LCTN (Figure S10, Supporting Information), implying that the kinetics of electrochemical oxidation of hydrogen was profoundly improved. This was mainly derived from the numerously exsolved nanocatalysts, yielding abundant active sites for the electrochemical reaction.^[^ ^8^, ^18^, ^20^, ^33^, ^35^ ^]^

As to the electrochemical oxidation of hydrogen (Figure S11, Supporting Information), generally, it can be expressed by the Kröger–Vink notation (1). When the nanocatalysts are uniformly dispersed on the surface of oxide support, dissociative adsorption of hydrogen molecule dynamically takes place on the surface of the nanocatalyst, enhancing the kinetics of adsorption and desorption processes. Thereafter, spillover of atomic hydrogen to the metal/support interface occurs in all directions. Concurrently, oxygen ions migrate to the interface through vacancy hopping followed by the reaction with spillover hydrogen to generate water. Subsequently, water desorbs from the surface. In fact, it has been demonstrated that the interface between nanoparticle and support is a preferable reaction region where the gas reactants, electrons, and ions can meet with each other, availing to the synergetic electrochemical reactions.^[^ ^9^ ^]^ Beneficially, the impressively exsolved nanocatalysts with anchored structure after thermal shock can extend this unique reaction region, facilitating the improvement of the electrochemical kinetics of hydrogen oxidation.^[^ ^22^, ^36^, ^37^, ^38^, ^39^ ^]^ Besides, there was no significant change on the electrochemical performance during the measurement (Figure 4h), suggesting a desirable stability of the cell. In a nutshell, thermal shock is a charming approach to rapidly fabricate NDM and tune the electrocatalytic activity of the host perovskite material.

(2) $$H_{2}\left(g\right)+O_{o}^{x}⇌H_{2}O\left(g\right)+V_{o}^{••}+2e^{-}$$

## 3 Conclusion

In summary, for the first time, we reported that by thermal shock technique, only ≈13 s (the fastest method as far as we know) was needed to fabricate the exsolution‐based NDMs, while it needed tens of hours for the conventionally furnace‐based methods. Benefitted by the strong driving force and short treating time, the NDM prepared by the thermal shock technique exhibited higher particle density and smaller particle size, yielding abundant active sites for the electrochemical reactions. A peak power density of 1003 mW cm^−2^ was attained at 900 °C using humidified hydrogen as the fuel. As compared to that of pristine LCTN, around 12‐fold enhancement of the cell performance was achieved. This work opens up a new way to fabricate NDMs in second scale, greatly improving the efficiency of preparing the exsolution‐based materials, which are very useful for—but not limited to—the electrochemical cells.

## 4 Experimental Section

### Preparation of Materials

La_0.43_Ca_0.37_Ti_0.94_Ni_0.06_O_3−_ *_*δ*_* oxide was prepared by the solid‐state reaction method. La_2_O_3_, CaCO_3_, TiO_2_, and NiO were used as the raw materials and weighed according to the stoichiometric ratio. The powders were fully mixed by a ball mill at 500 rpm for 24 h using zirconia balls with different size. During the ball mill, ethanol was used as the media. After stirring and evaporating of the ethanol at 70 °C, the collected powders were calcined at 1100 °C for 20 h and 1350 °C for 20 h, respectively. For de‐agglomeration, subsequently, the as‐synthesized powders with target phase were ball milled, resulting in a different size of the particles.

### Cell Fabrication

The as‐synthesized powders were mixed with terpinol, KD1, and polyvinyl butyral to prepare the ink, which was screen‐printed (325 mesh) on the center of the YSZ electrolyte. Then, the ink of (La_0.8_Sr_0.2_)_0.95_MnO_3_ (LSM) and YSZ with a weight ratio of 50:50 was screen‐printed on the other side of YSZ symmetrically. Gold mesh was attached on the top of the electrode by gold paste and applied as the current collector.

### Characterization

Crystal structure of the as‐prepared powders was investigated by the Bruker D8 Advance X‐ray diffractometer. X'Pert HighScore Plus software was used to refine the collected XRD data. SEM FEI Quanta 600F was carried out to check the microstructure. For thermal shock, the as‐prepared LCTN powders were first converted into ink which was then screen‐printed on the surface of the YSZ electrolyte. Subsequently, the as‐prepared cell was transferred into a home‐made chamber. Before thermal shock, the home‐made chamber was highly vacuumed first and then 5%H_2_/N_2_ was introduced at a flow rate of 50 mL min^−1^. After flushing about 15 min, the thermal shock was carried out. During the thermal shock, a customized carbon paper was used as the heating source, and the current was supplied by a power supply which could be remotely controlled by the designed software. The temperature was estimated through fitting the ultraviolet‐visible (UV‐vis) spectra collected by a high‐speed camera. An electrochemical workstation (Solartron 1260/1287) was applied to record the EIS. The scanned frequency range was from 10^5^ to 10^−1^ Hz, and the AC amplitude was 10 mV. Aremco Ceramabond 552 was used to seal the cell to the home‐made setup. During the measurements, thermocouple was used to monitor the environmental temperature. Linear sweep voltammetry was adopted to collect the *I*–*V* curves, the voltage was scanned from OCV to 0 V. Hydrogen was introduced into the anode chamber at a flow rate of 100 mL min^−1^ and the cathode was placed in the ambient air.

### Statistical Analysis

For SEM statistical analysis, a relative flat surface of the sample was selected and used. Commercial software Nano Measure1.2 was applied for measuring the size of individual particles and counting the particle number from the SEM results. Thus, a size distribution was obtained and plotted by using commercial software Origin85. The results were presented as mean ± SD (standard deviation). The particle density was calculated from *N*/*S*, where *N* is the particle amount and *S* is the surface area.

## Conflict of Interest

The authors declare no conflict of interest.

## Author Contributions

Z.S. and W.W.F. contributed equally to this work. Z.S. and W.W.F. conceived the concept and designed the experiments. Z.S. and W.W.F. prepared the materials and fabricated the cells, as well as performed the tests. Z.S. and W.W.F. drafted the manuscript. Z.S., W.W.F., and Y.B. discussed the data and revised the manuscript.

## Supporting information

Supporting Information

## Data Availability

The data that support the findings of this study are available from the corresponding author upon reasonable request.
