# Supplementary material for: A Flexible Method to Fabricate Exsolution‐Based Nanoparticle‐Decorated Materials in Seconds
Source: Adv Sci (Weinh). 2022 Feb 20;9(12):2200250. doi: 10.1002/advs.202200250 (PMC9036016; doi:10.1002/advs.202200250)
Supplement: Supplementary file 1 — Supporting Information [file ADVS-9-2200250-s001.pdf]

Supporting Information

A flexible method to fabricate exsolution-based nanoparticle-decorated materials in seconds

Zhu Sun,[1,4] Weiwei Fan,*[,2,4], Yu Bai*[,3]

[1]State Key Laboratory of Electrical Insulation and Power Equipment, Xi'an Jiaotong University, Xi'an 710049, People's Republic of China

[2]Department of Nuclear Science and Engineering, Massachusetts Institute of Technology, Cambridge 02139, USA

[3]State Key Laboratory for Mechanical Behavior of Materials, Xi'an Jiaotong University, Xi'an 710049, People's Republic of China

[4]These authors contributed equally to this work.

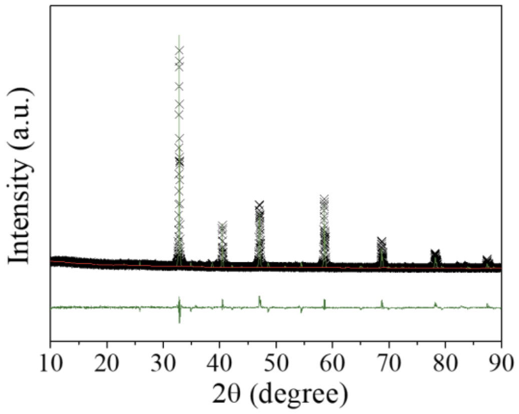

Figure S1. Rietveld refinement XRD profile of the as-synthesized LCTN oxide.

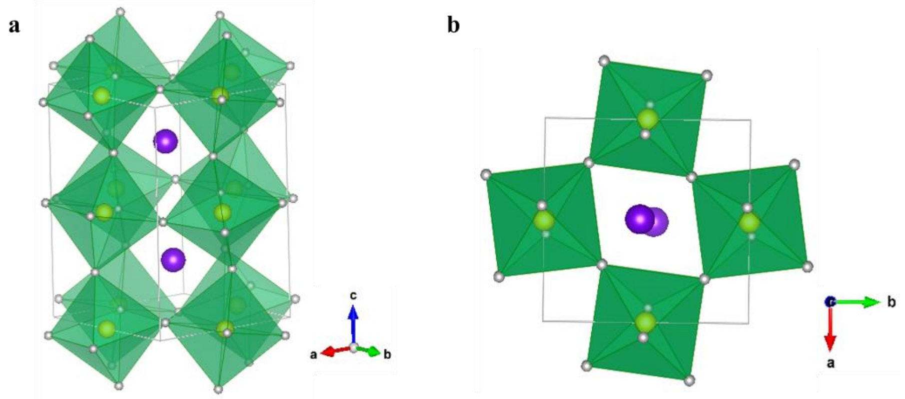

Figure S2. **a,b,** Crystal structural of LCTN oxide at different views. Purple spheres denote La/Ca, yellow spheres denote Ti/Ni, grey spheres denote O.

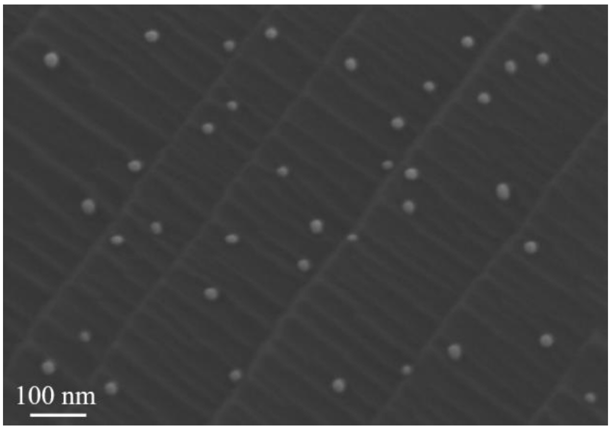

Figure S3. Enlargement view of Figure 2b.

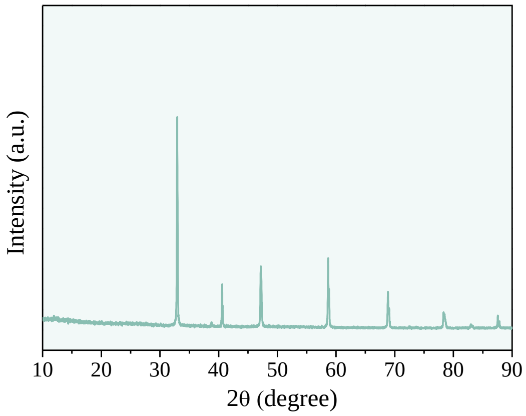

Figure S4. XRD profile of the as-synthesized LCT oxide.

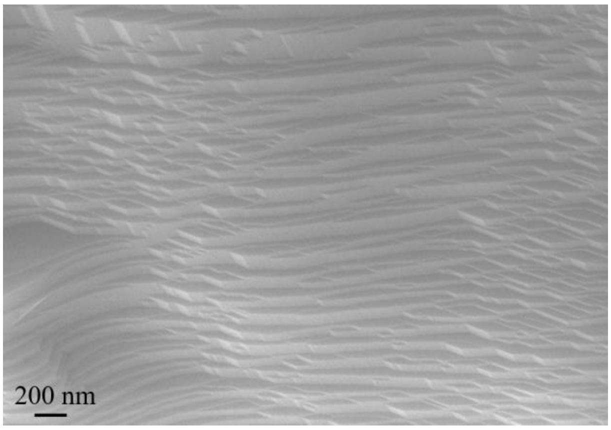

Figure S5 SEM image of LCT perovskite after thermal shock.

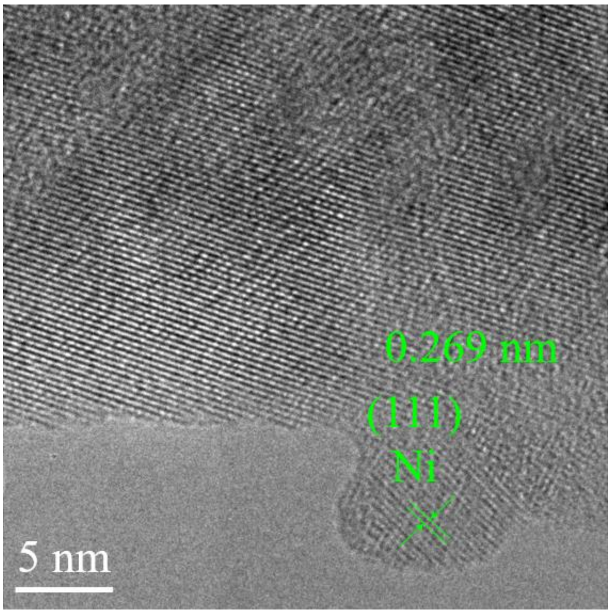

Figure S6. HR-TEM image of the exsolved particle after thermal shock.

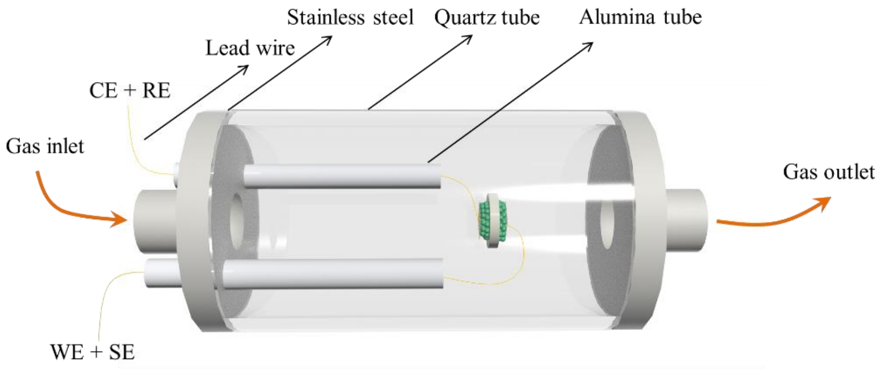

Figure S7. Schematic of the home-made setup for the half cell performance test.

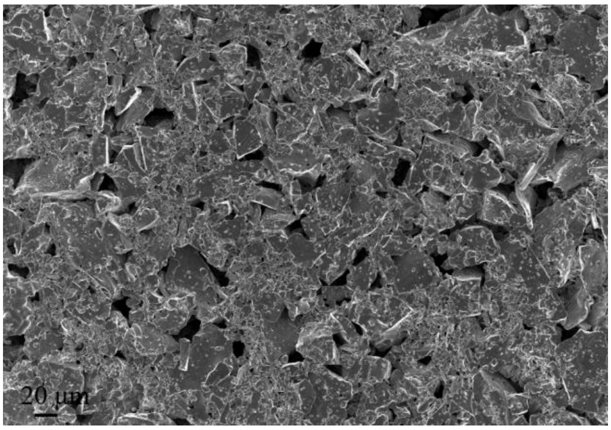

Figure S8. SEM image of the LCTN anode after thermal shock.

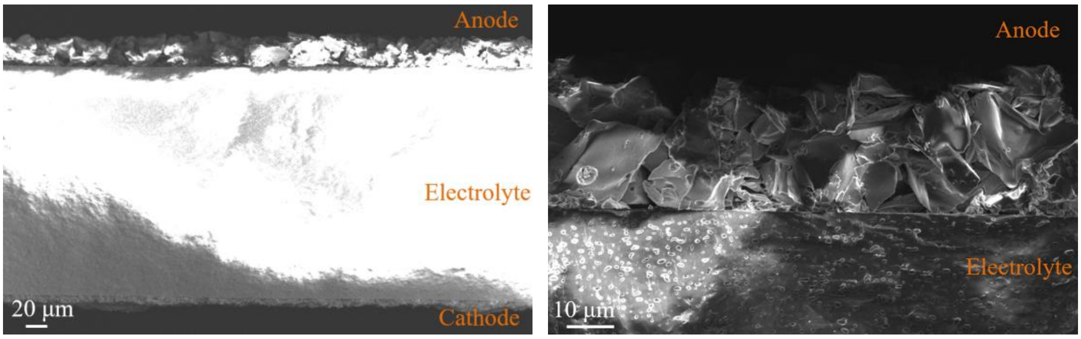

Figure S9 SEM image of the full cell after thermal shock.

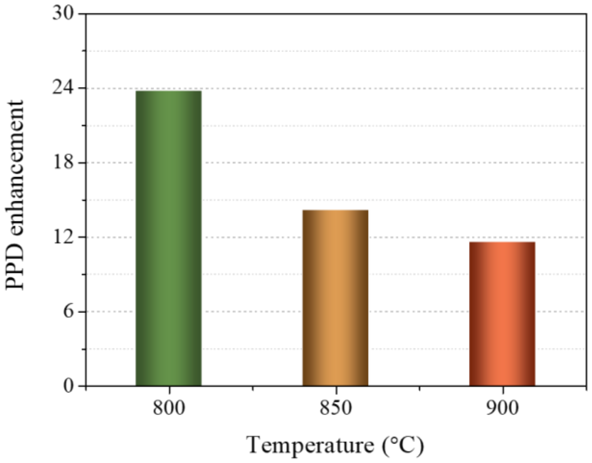

Figure S10. PPD enhancement of the thermal shock LCTN as compared to that of pristine one.

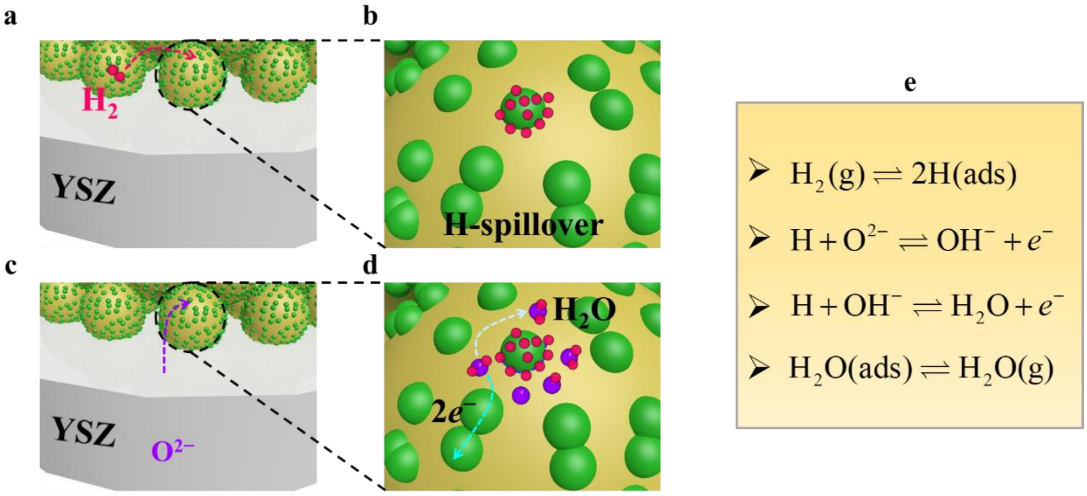

Figure S11. Processes of electrochemical oxidation of H_2, including H-spillover, water formation and desorption.

Table S1. Refined structural parameters for LCTN obtained by fitting of powder XRD data at room temperature.

Atoms, sites	Parameters	LCTN
	Space group	Pbnm (62)
	a (Å)	5.4643(4)
	b (Å)	7.7337(4)
	c (Å)	5.4632(7)
	V (Å^3)	230.87
La, 4c	*x*	0.4670
	y	0.2500
	z	0.0072
	Occupancy	0.43
Ca, 4c	*x*	0.4670
	y	0.2500
	z	0.0072
	Occupancy	0.37
Ti, 4a	*x*	0
	y	0
	z	0
	Occupancy	0.94
Ni, 4a	*x*	0
	y	0
	z	0
	Occupancy	0.06
O1, 4c	*x*	0.5107
	y	0.2500
	z	0.5722
	Occupancy	1
O1, 8d	*x*	0.2158
	y	0.0346
	z	0.2826
	Occupancy	1
	R_{wp}	14.14
	R_p	10.58
	χ^2	3.70
